# First Detection and Genome Sequencing of SARS-CoV-2 Lambda (C.37) Variant in Symptomatic Domestic Cats in Lima, Peru

**DOI:** 10.3389/fvets.2021.737350

**Published:** 2021-09-17

**Authors:** Francesca Schiaffino, Cusi Ferradas, Luis M. Jara, Guillermo Salvatierra, Alejandra Dávila-Barclay, Camila Sanchez-Carrion, Alexandra Ulloa, Lucero Mascaro, Monica J. Pajuelo, Luis Guevara Sarmiento, Manolo Fernandez, Mirko Zimic

**Affiliations:** ^1^Facultad de Medicina Veterinaria y Zootecnia, Universidad Peruana Cayetano Heredia, Lima, Peru; ^2^Unidad de Investigación en Enfermedades Emergentes y Cambio Climático (Emerge), Facultad de Salud Pública y Administración, Universidad Peruana Cayetano Heredia, Lima, Peru; ^3^Clínica Veterinaria Gatuario, Lima, Peru; ^4^Laboratorio de Microbiología Molecular, Facultad de Ciencias y Filosofía, Universidad Peruana Cayetano Heredia, Lima, Peru; ^5^Farmacologicos Veterinarios (FARVET), Chincha, Peru; ^6^Laboratorio de Bioinformatica, Biologia Molecular y Desarrollos Tecnológicos, Facultad de Ciencias y Filosofía, Universidad Peruana Cayetano Heredia, Lima, Peru

**Keywords:** COVID-19, one health, SARS-CoV-2, surveillance, felis catus

## Abstract

The role of domestic cats in the dynamics of SARS-CoV-2 remains poorly characterized, especially in epidemiologic contexts of countries with high viral transmission. Here, we report the first evidence of SARS-CoV-2 Lambda variant of interest in symptomatic domestic cats whose owners were diagnosed with COVID-19 in Lima, Peru, providing evidence that transmission of this new variant in domestic cats is occurring. More epidemiological studies are required to further characterize the role of domestic animals in the transmission dynamics of SARS-CoV-2.

## Introduction

SARS-CoV-2 transmission to domestic animals, mainly dogs and cats, has been reported in various countries, such as the United States, Brazil, France, Switzerland, Chile, China, Belgium, among others (1–7). Studies have provided evidence for human to animal transmission, as well as experimental animal to animal transmission of the virus (8). However, zoonotic transmission from domestic animals back to humans has not been evidenced (9). Minks and other animals could act as potential viral reservoirs where genomic mutations or variants could arise (10). If SARS-CoV-2 were to cross the animal—human interface, such as it is hypothesized it did originally (11–13), and with minks (14), domestic cats could also serve as sources and sinks of relevance in human transmission.

Latin America has been one of the heavily COVID-19 impacted regions of the world, with Peru experiencing 6,405 cases per 100,000 individuals and 600 deaths per 100,000 individuals until late June, 2021 (15). The Lambda variant (C.37) was first reported in December 2020 in Peru, and has since spread in South America (16) and expanded to other continents, currently accounting for 16.9% (242/1436) of genomes available at Global Initiative on Sharing All Influenza Data (GISAID) (17). Importantly, it is currently considered a variant of interest (VOI) by the World Health Organization (18). This variant is a sub lineage of the B1.1.1 lineage and it presents a deletion in the ORF1a gene and multiple non-synonymous mutations in the spike gene (19).

The extent to which domestic animals have been affected by SARS-CoV-2 in this same region is unknown. In Lima, Peru, domestic cats can have both indoor and outdoor lifestyles, and they generally have intense human contact. Since the first COVID-19 wave, evidence of SARS-CoV-2 infection in domestic cats has not been reported in this area, nor have studies investigating the role of domestic cats as potential viral reservoirs been conducted. Here we report genomic evidence of the SARS-CoV-2 VOI Lambda in three symptomatic domestic cats living in households with humans with a history of COVID19 disease, in Lima, Peru.

## Methods

### Sample Collection and Preliminary Testing

Between March and May 2021, domestic cats presenting to a small animal veterinary clinic in Lima, Peru with respiratory or gastrointestinal symptoms and whose owners reported a history COVID-19 illness among humans living in the same household were further screened for SARS-CoV-2 infection. Specifically, attending veterinarians asked the cat owner about COVID-19 disease in any household member as part of the regular COVID-19 preventive protocol and diagnostic algorithm of the veterinary clinic. The attending veterinarian had no further information regarding the human case of COVID-19 within the household, and limited contact with owners with a history of potential COVID-19 exposure. Clinical exams included complete blood count (CBC) (IDEXX ProCyte Dx Hematology Analyzer) and thoracic radiographic imaging. Given a recent history of COVID-19 in owners, veterinarians recommended a lateral flow antigenic testing (COVID-19 Antigen Rapid Test Kit, Konsung, China) and molecular test for SARS-CoV-2. Pharyngeal swabs were obtained from animals and placed in a viral transport medium (CITOSWAB VTM, WellKang, UK) for further molecular diagnostic analysis.

### SARS-CoV-2 RT-qPCR

Samples underwent RT-qPCR diagnosis as described previously (20). Total viral RNA was extracted using the QIAamp RNA Mini Kit (QIAGEN, Hilden, Germany) according to the manufacturer's instructions in a BSL-3 laboratory. qPCR reaction mixtures of 20 uL, including 5 uL Master Mix (TaqMan Fast Virus 1-Step Master Mix, Applied Biosystems, Massachutts, USA), forward and reverse primers for nucleocapsid gene (Fw: 5′- AAATTTTGGGGACCAGGAAC-3′, Rv: 5′-TGGCACCTGTGTAGGTCAAC−3′) at 10 uM, probe (FAM-ATGTCGCGCCATTGGCATGGA-BHQ) at 10 uM, 5 uL of RNA template and Nuclease free-water (Invitrogen, Massachusetts, USA). Negative template controls (RNAse free water) were included in the amplification reaction. Reaction mixtures in duplicates were placed in a 96-well plate and amplified using a StepOnePlus real time PCR system (Applied Biosystems, Massachusetts, USA). Amplification conditions included 50°C for 5 mi, 95°C for 20 s, 45 cycles of 95°C for 3 s, 60°C for 30 s. Samples with a Cycle threshold (Ct) of <40 were considered positive and samples with a Ct of 35 or less underwent Next-generation sequencing (NGS).

### Sequencing and Bioinformatic Analysis

SARS-CoV-2 positive RNA samples underwent reverse transcription, target amplification, library preparation and pooling using the Illumina COVIDSeq Test (Illumina, Inc., San Diego, CA, USA) workflow, and sequenced on an Illumina MiniSeq instrument with a MiniSeq System Mid-Output Kit using a negative amplification control and generating 150 bp paired-end reads. Raw reads were processed through the Illumina DRAGEN COVID Lineage v3.5.3 BaseSpace App. Consensus sequences were generated using the alignment to SARS-CoV-2 reference genome (NC_045512.2) with a coverage threshold of 20, virus detection threshold of 5, and default parameters. Lineage analysis was performed using Pangolin v3.0.3 (github.com/cov-lineages/pangolin) available at DRAGEN COVID Lineage App following the Pango nomenclature (20). NextClade v1.5.0 web tool (clades.nextstrain.org/) was employed for clade determination and mutation calling.

The raw sequencing reads of SARS-CoV-2 have been submitted to National Center for Biotechnology Information (NCBI) under BioProject number PRJNA743043, (https://www.ncbi.nlm.nih.gov/bioproject/?term=PRJNA743043) and the draft genome assemblies were deposited in GenBank under accession numbers MZ496613, MZ496614 and MZ496616, and, submitted to the GISAID EpiCoV database under accession IDs EPI_ISL_2791488, EPI_ISL_2791489, and EPI_ISL_2791490.

## Results

Antigen testing was performed on all cats except Cat 4 due to owners' decision. Lateral flow tests of all four cats were positive. Five cats were screened for SARS-CoV-2, of which four were positive via qPCR amplification. SARS-CoV-2 was confirmed in three animals (Cat 1, Cat 2, and Cat 3) using NGS. Demographic and clinical characteristics, including vital signs, thoracic imaging results of each animal are presented in Table 1. CBC showed thrombocytopenia and eosinopenia in Cat 3 and Cat 4, respectively, and no other significant alterations in the rest of the animals. For the full set of values from the CBC see Supplementary Table 1. Cat 1 and Cat 2 lived within the same household among 4 other animals of which only them presented respiratory symptoms. Cat 3 lived with another cat in the same household, who showed only slight sneezing, yet this animal was not tested for SARS-CoV-2.

**Table 1:** Characteristics of four domestic cats with suspected SARS-CoV-2 infection.

	**Cat 1**	**Cat 2**	**Cat 3**	**Cat 4**	**Cat 5**
Sex	Male (neutered)	Female (spayed)	Female (intact)	Male (neutered)	Male (neutered)
Age (years)	3.5	3.5	5	5.5	2
Breed	Short-haired	Short-haired	Short-haired	Short-haired	Short-haired
Vital signs upon examination	Heart rate: 200 bpm Respiratory rate: 36 bpm Temperature: 38.6^o^C	Heart rate: 160 bpm Respiratory rate: 40 bpm Temperature: 39.1^o^C	Heart rate: 196 bpm Respiratory rate: 38 bpm Temperature: 38.9^o^C	Heart rate: 200 bpm Respiratory rate: 64 bpm Temperature: 36.9^o^C	Heart rate: 200 bpm Respiratory rate: 36 bpm Temperature: 38.6^o^C
Date of sampling	4/26/21	4/26/21	5/2/21	3/25/21	5/18/21
Symptoms	Sneezing, depression, cough	Sneezing, depression, cough, tachypnea	Cough	Vomiting, anorexia, dyspnea	Cough, dyspnea
Lateral thoracic radiographic imaging	Thickened bronchial walls	Thickened bronchial walls	Mildly thickened bronchial walls	Not Performed	Thickened bronchial walls
Patient outcome	−1 month after initial symptoms, mild cough. - 2 months after initial symptoms, no symptoms.	−1 month after initial symptoms, cough, and sneezing. - 2 months after initial symptoms, no symptoms.	−1 month after initial symptoms, mild cough. - 2 months after initial symptoms, no symptoms.	- Presumptive chronic pancreatitis with dyspnea. - Euthanized.	- Presumptive pneumonia. - 1 month after initial symptoms, no symptoms.
Owner(s) with confirmed COVID19 disease	Yes (1 to 2 weeks prior to cat symptom onset)	Yes (1 to 2 weeks prior to cat symptom onset)	Yes (1 week prior to cat symptom onset)	Yes (time not available)	Yes (1 month prior to cat symptom onset)
Sample type	Pharyngeal	Pharyngeal	Pharyngeal	Pharyngeal	Pharyngeal
COVID-19 antigen detection	Positive	Positive	Positive	Not performed	Positive
qRT-PCR cycle threshold	19.8	25.8	25.3	37	Negative
Sequencing analysis (lineage)	Lambda (C.37 variant)	Lambda (C.37 variant)	Lambda (C.37 variant)	None	None

*Hear rate: bpm, beats per minute; respiratory rate: bpm, breaths per minute*.

NGS confirmed the detection of SARS-CoV-2 genomes (2685.3 × mean coverage) from the three sequenced cat pharyngeal samples. All genomes were classified as the previously reported clade 21G, and lineage C.37 (19) VOI Lambda (21) containing the previously reported deletion (S:Δ247-253, located at the N-terminal domain) (22) and seven nonsynonymous mutations in the Spike gene (G75V, T76I, D253N, D614G, L452Q, F490S, and T859N).

## Discussion

This study is the first report of SARS-CoV-2 in domestic cats with symptomatic disease in Peru. It also confirms that domestic cats can acquire the SARS-CoV-2 Lambda VOI, and it is significant because it is the first report of such an event. SARS-CoV-2 has also been reported in South American pets, yet only sequenced in cats in Chile, Argentina (Pango lineage B.1.499, GISAID Accession Number 1914577), Colombia (Pango lineage B.1.111, GISAID Accession Number 2339859) and Brazil (GISAID Accession Numbers 848083, 848071, 848070) (4, 16). Although the nature of this exploratory study did not allow for samples from humans in close contact with the cats to be analyzed, human to animal transmission of SARS-CoV-2 is hypothesized. Additionally, serum-neutralizing antibodies against the receptor binding domain of the viral spike protein of SARS-CoV-2 have also been detected in cats living with owners with a history of COVID-19 disease in Lima demonstrating immune response from viral exposure (23). These findings as well as the potential higher infectivity of the Lambda variant further highlight the importance of initiating SARS-CoV-2 surveillance in domestic animals in Latin American countries where viral transmission is high (24, 25).

These findings capitalized passive surveillance initiated by a small animal veterinary clinic in Lima, Peru. The study is limited by the small number of animals included in the analysis, limited clinicopathological information available from these cats, as well as the lack of testing in asymptomatic animals. However, our group recently reported evidence of serum neutralizing antibodies against SARS-CoV-2 in asymptomatic cats from owners with a history of COVID-19 (23). Additionally, there is evidence to show that infection in domestic animals can be accompanied with heterogenous clinical signs and hematologic abnormalities, including the absence of these (1). The study is also limited by lack of additional ancillary testing to rule out feline specific pathogens associated with upper respiratory disease. As a result, due to the nature of this investigation the clinical signs observed in cats cannot be causally attributed to SARS-CoV-2. Future investigations should explore the epidemiologic role of cats within communities with high levels of transmission as well as investigate if specific variants of SARS-CoV-2 are more likely to infect and transmit among cats and produce clinical disease among them. This will not only aid in determining the role domestic animals play as possible reservoirs of SARS-CoV-2, but also provide evidence that helps educate pet owners and train veterinarians to adequately improve differential diagnosis and manage these cases. Veterinarians should take precautionary measures when handling symptomatic cats with a confirmed COVID-19 positive contact, and owners should isolate themselves from their domestic animals when infection is suspected to prevent human to animal transmission of SARS-CoV-2. Although animal to human transmission has not been reported from infected cats, infection in a new host provides opportunities for evolutionary changes in a virus, with potential consequences for transmissibility and pathogenicity in humans and effectiveness of a vaccine (26).

## Conclusion

This report provides the first genomic evidence of SARS-CoV-2 VOI Lambda in symptomatic domestic cats in Lima, Peru. It is crucial to continue investigating the genomic epidemiology of SARS-CoV-2 in domestic animals yet under robust epidemiological designs that elucidate the role of domestic animals in the transmission dynamics or reservoir of new variants of this virus.

## Data Availability Statement

The datasets presented in this study can be found in online repositories. The names of the repository/repositories and accession number(s) can be found in the article Methods section.

## Ethics Statement

Ethical review and approval was not required for the animal study because the study only included owners who attended the small animal veterinary clinic to request standard medical care for their animals. Given a history of COVID-19 in owners (self-reported by them), veterinarians requested SARS-CoV-2 diagnostics. Samples were sent to Universidad Peruana Cayetano Heredia for diagnostic testing with the consent of the owner. Samples were de-identified, and no data on owner demographic and clinical characteristics were obtained. Written informed consent for participation was not obtained from the owners because of the same reasons stated previously.

## Author Contributions

FS, CF, and LJ: conceptualization, formal analysis, investigation, roles, writing—original draft, writing—review and editing, and methodology. GS, AD-B, LM, and MJP: methodology, data curation, and writing—review and editing. CS-C and AU: investigation and writing—review and editing. LG: conceptualization, writing—review and editing, and methodology. MF: conceptualization, funding acquisition, and writing—review and editing. MZ: conceptualization, methodology, formal analysis, funding acquisition, and writing—review and editing. All authors contributed to the article and approved the submitted version.

## Conflict of Interest

The authors declare that the research was conducted in the absence of any commercial or financial relationships that could be construed as a potential conflict of interest.

## Publisher's Note

All claims expressed in this article are solely those of the authors and do not necessarily represent those of their affiliated organizations, or those of the publisher, the editors and the reviewers. Any product that may be evaluated in this article, or claim that may be made by its manufacturer, is not guaranteed or endorsed by the publisher.
