# Supplementary material for: First Detection and Genome Sequencing of SARS-CoV-2 Lambda (C.37) Variant in Symptomatic Domestic Cats in Lima, Peru
Source: Front Vet Sci. 2021 Sep 17;8:737350. doi: 10.3389/fvets.2021.737350 (PMC8484519; doi:10.3389/fvets.2021.737350)
Supplement: Supplementary file 1 [file Table_1.DOCX]

**Supplementary Table 1**. Complete blood count values of the five domestic cats screened for SARS-CoV-2

| **Item** | **Cat 1** | **Cat 2** | **Cat 3** | **Cat 4** | **Cat 5** | **Reference Value** |
| --- | --- | --- | --- | --- | --- | --- |
| RBC | 8.74 M/ul | 10.11 M/ul | 10.67 M/ul | 9.85 M/ul | 9.67 M/ul | 6.54-12.20 M/ul |
| Hematocrit | 40.90% | 41.90% | 47.70% | 46.10% | 47.60% | 30.3 - 52.3% |
| Hemoglobin | 12.5 g/dL | 13.6 g/dL | 13.5 g/dL | 14.3 g/dL | 15.6 g/dl | 9.8 - 16.2 g/dL |
| MCV | 46.8 fL | 41.4 fL | 44.7 fL | 46.8 fL | 49.2 fL | 35.9 - 53.1 fL |
| MCH | 14.3 pg | 13.5 pg | 12.7 pg | 14.5 pg | 16.1 pg | 11.8 - 17.3 pg |
| MCHC | 30.6 g/dL | 32.5 g/dL | 28.3 g/dL | 31.0 g/dL | 32.8 g/dL | 28.1 - 35.8 g/dL |
| RDW | 22.20% | 25.20% | 27.50% | 25.40% | 23.50% | 15.0 - 27.0% |
| Reticulocytes | 26.2 K/ul | 17.2 K/ul | 43.7 K/ul | 16.7 K/ul | 31.9 K/ul | 3.0 -50.0 K/uL |
| WBC | 7.45 K/ul | 5.3 K/ul | 4.14 K/ul | 8.3 K/ul | 7.08 K/ul | 2.87 - 17.02 K/uL |
| Neutrophils | 4.06 K/ul | 3.38 K/ul | 2.36 K/ul | 6.55 K/ul | 3.22 K/ul | 2.30 - 10.29 K/uL |
| Lymphocytes | 2.65 K/ul | 1.41 K/ul | 1.42 K/ul | 1.28 K/ul | 2.9 K/ul | 0.92 - 6.88 K/uL |
| Monocytes | 0.40 K/ul | 0.23 K/ul | 0.11 K/ul | 0.34 K/ul | 0.38 K/ul | 0.05 - 0.67 K/uL |
| Eosinophils | 0.33 K/ul | 0.27 K/ul | 0.22 K/ul | 0.07 K/ul | 0.52 K/ul | 0.17 - 1.57 K/uL |
| Basophils | 0.01 K/ul | 0.01 K/ul | 0.03 K/ul | 0.06 K/ul | 0.06 K/ul | 0.01 - 0.26 K/uL |
| Platelets | 475 K/ul | 248 K/ul | 66 K/ul | 371 K/ul | 441 K/ul | 11.4 - 21.6 K/uL |

Platform and Assay: IDEXX ProCyte Dx Hematology Analyzer
